# Health Tract, No. 159

**Published:** 1863-08

**Authors:** 


HEALTH TRACT, NO. 159.

### CIVIL AND MILITARY DEATH-RATE.

The only son of a New-York capitalist entered the army as a private, aged eighteen. In an outburst of youthful generosity he resigned his own shelter in behalf of a poor soldier and slept in the rain, with his feet resting in a little stream of water, and died in a few days. In a recent march of the Army of the Potomac one thousand soldiers were sun-struck, of which number about one hundred died within twenty-four hours. In both cases, the son and the soldiers died from ignorance. A few green leaves or a silk handkerchief in the hat would have prevented those sun-strokes; had the young man known the necessity of sleeping with dry, warm feet, he would have lived. These two items, with more than a hundred others, are detailed in our twenty-five cent book on "Soldier Health;" the object being to name in each rule, in as few and as plain words as possible, the means of guarding against sickness, of remedying disease, and treating various kinds of wounds with the means which any soldier *is sure to have about him.* The book does not presuppose the sick or wounded man is in a populous city or on the floor of a drug-store; but takes it for granted that he is alone in the woods, or wounded and lost or forsaken on the battle-field, and shows him how, with his ramrod or a stick and a strip of his shirt, he can staunch the severest wound; or how, with a little powder, he may avert instant death; or with a bit of cloth can arrest one of the most fearful of diseases. Short explanations of the reason of these things are given, so as to impress the idea on his mind, and thus carry the contents of the little work in his head; for even a watch-fob volume is an incumbrance on a march or in a fight. Intelligence is the best life-preserver. The largest city in the civilized world is healthier than its surrounding agricultural district. The aristocratic regiment of New-York was gone a month or two to the war, and returned with the loss of but one man in eight hundred, and he died of heart disease of long duration. Of some ninety persons who went to the army in various capacities from one church, no more died of all causes than among an equal number at home. These things seem to show that intelligence, especially connected with social elevation, are promotive of health; and considering that an active, out-door soldier's life works disease out of the system, especially where there is no addiction to social vices, there is good ground for believing that even with the addition of the casualties of war there need not be any more deaths in a given time among a given number of men than there would have been in the same men had they remained at home. With these views, every parent who sends a son or relative to the war, should first place in his hand some succinct, reliable little book; not to be taken along so much, but to be read over, mastered, and remembered, so that he may know how to act in an emergency; how to act in case of being wounded or taken sick in some desolate place. Attention to this suggestion might save many a life. The lowest death-rate reported from a civilized community is twelve out of a thousand in one year. It is twenty-two per thousand for all England; twenty-four for the United States, and twenty-eight cases of sickness for each death.

